# Individualism, Collectivism, and Allocation Behavior: Evidence from the Ultimatum Game and Dictator Game

**DOI:** 10.3390/bs13020169

**Published:** 2023-02-14

**Authors:** Jingjing Jiao, Jun Zhao

**Affiliations:** School of Economics and Management, South China Normal University, Guangzhou 510006, China

**Keywords:** individualism, collectivism, ultimatum game, dictator game, allocation behavior

## Abstract

Studies have demonstrated the influence of the cultural values of individualism and collectivism on individuals’ economic behavior (e.g., competition and trade). By using individualistic and collectivistic texts to prime participants’ minds in a lab experiment, we investigated the impact of the cultural values of individualism and collectivism on allocation behavior in an ultimatum game (UG) and dictator game (DG). In the dictator game, we found that participants in the collectivism-priming condition reported a slightly higher mean offer than in the individualism-priming condition, and participants had an average higher acceptance rate of the proposers’ offer in the collectivism-priming (vs. individualism-priming) condition in the ultimatum game. Our findings suggest that participants exhibit more altruistic allocation behavior and are more tolerant of unfair allocation behavior after being primed by the collectivistic (vs. individualistic) texts. In comparison with participants who did not undergo initiation, we also found that Chinese participants who had been influenced by collectivist values for a long time remained unaffected after the initiation of collectivism, but shifted their allocation behavior (i.e., showed decreased altruistic allocation behavior and reduced tolerance of unfair allocation behavior) when individualism was primed.

## 1. Introduction

Among studies of how culture shapes human behaviors, the question of how culture affects people’s economic behavior is immensely influential [1,2]. In this paper, we concentrated on one critical dimension of culture, individualism–collectivism culture values [3,4,5,6]. Experimental economics research has found that individualism and collectivism influence people’s competition behavior [7] and trade behavior [8]. However, cultural variation in allocation behavior has not been sufficiently examined. People engage in a variety of allocation decisions in daily life (e.g., company earnings and household assets), where their strategies in regard to allocation decisions are influenced by culture. The primary objective of this paper is to examine the role of individualism and collectivism in allocation behavior. To this end, we choose the ultimatum game (UG) [9] and dictator game (DG) [10] to capture people’s allocation behavior. 

### 1.1. Individualism-Collectivism Culture Values

The key difference between individualism and collectivism is how people view themselves in relation to others. In a collectivist culture, the self is interdependent on members of the group, and collectivists place group concerns (e.g., group harmony and cohesion) above personal concerns (e.g., self-enhancement); in contrast, the self in an individualist culture is autonomous and independent of the group, and individualists place individual concerns above those of the group [4,5,6,11]. 

In this paper, we used cultural priming techniques to activate either individualism or collectivism. Cultural psychologists have developed cultural priming techniques to manipulate cultural value systems within individuals. Commonly used individualism–collectivism priming tasks include the pronoun circling task [12,13], Sumerian warrior story [13,14], similarities vs. differences with family and friends task (SDFF) [14], scrambled sentence task [15,16], group instantiation [17] and group imagination [18]. This study uses two of them, namely the pronoun circling task and group imagination. Meta-analysis of the individualism and collectivism priming literature showed that the results are robust across priming methods and consistent in direction with cross-national effects, which means that, depending on the situational context, human behavior could be switched over between individualism and collectivism [6]. Existing studies priming individualism and collectivism have found that the values, interpersonal relationships, self-concept, and cognitive abilities of subjects are greatly affected after priming [6,13,14].

### 1.2. Ultimatum Game and Dictator Game

The ultimatum game [9] is an economic game played by two individuals, a proposer, and a responder. The proposer is given a certain amount of money by the experimenter and is asked to propose a way of dividing the money between himself and the responder. The responder then has to choose to accept or reject the offer. If the responder accepts the offer, both the proposer and the responder earn money as specified by the proposer. However, if the responder rejects the offer, neither player receives any money. The dictator game [10] was designed to control strategic behavior in the ultimatum game; the responder has no power to punish the proposer or to refuse the money. 

According to classical economic theory, the proposer in both games should give the responder the smallest positive amount of money to maximize his or her payoff. However, various studies have found that proposers generally give approximately 40% of the sum to the responders in the ultimatum game and approximately 30% of the total amount to the responders in the dictator game [19,20]. Previous studies showed that responders in the UG are likely to reject unfair offers, especially for offers below 20% of the total in order to punish the unfair proposer by ensuring that they get nothing [9,21,22,23].

Allocation behaviors in this paper can be divided into two main categories, namely proactive behaviors (offer in DG, DG) and reactive behaviors (accept or reject in UG). In the UG, the offer combines two motivations: concerns for fairness and the fear of payback [24]; high offers in UG may not be only due to pure altruistic motivation, but also strategic consideration to maximize their benefits [25]. In the DG, the offer is not affected by strategic considerations (e.g., the fear of payback), and to measure altruistic allocation behavior [26]. The rejection of unfair offers in UG can be regarded as evidence of inequity aversion [27].

### 1.3. Individualism-Collectivism Culture Values and Allocation Behavior

The extant cross-country/cross-culture literature has found that group or country differences are reflected in the allocation behavior in the ultimatum and dictator games [1,2,19,20,28,29,30,31,32,33,34,35,36,37]. For example, Henrich et al. investigated how the social and economic environment as well as cultural differences can shape the participant’s behavior in experimental games [1,2]. Engel summarized the evidence from the past 25 years of dictator experiments and addressed the issue of the relationship between DG offers and the level of development of a country [19]. However, the causal interpretation of cultural differences remains unclear, as cultures differ in numerous dimensions. Oosterbeek et al. [20] conducted a meta-analysis of 37 papers with 75 results from the ultimatum game. They related subjects’ behavior to specific cultural traits [38,39], and found that the individualism index had a nonsignificant effect on offers or reject rates. Zhu et al. [40] found that the size of offers in the ultimatum and dictator games is an increasing function of the number of years Chinese citizens experienced the Maduringera (“planned economy”), which indicates that collectivism values influence people’s allocation behavior.

### 1.4. Present Study

Our study suggests that participants exhibited more altruistic allocation behavior in DG and were more tolerant of unfair allocation behavior in UG after being primed by the collectivistic (vs. individualistic) texts. The paper makes the following contributions. First, most experimental economics studies of collectivism and individualism have employed field experiments [7,41]. Our experiments provide a useful tool to bring the study of the connection between cultural values and allocation behavior into a laboratory setting. Next, we provide evidence of causality in the relationship between individual-level individualism–collectivism and allocation behavior by priming subjects with individualism–collectivism values and observing the effects. Finally, our findings are valuable in explaining the differences in cross-country/cross-cultural allocation behavior in the ultimatum and dictator games.

The rest of the paper is organized as follows. Section 2 describes the experimental details and procedures. Section 3 presents the data and analysis of the present study, followed by the results. Section 4 outlines the limitations of the study and future research directions. Section 5 concludes this study. 

## 2. Materials and Methods

### 2.1. Participants

A total of 240 Chinese subjects, comprising 118 males and 122 females between 17 and 28 years of age, participated in the experiment. Subjects were recruited from among college students at South China Normal University via a social media platform. We balanced the number of men and women in each subject group. 

### 2.2. Procedure

To verify the single priming effect, we added the no-priming condition. The experiment included 10 sessions with 24 subjects each. In each session, subjects ranged from one to eight in the collectivism-priming treatment, from 9 to 16 in the individualism-priming treatment, and from 17 to 24 in the no-priming treatment. All participants were randomly assigned to collectivism-priming, individualism-priming, or no-priming conditions. No participant attended more than one treatment (with no more than one session per subject). Experiments were conducted at South China Normal University between January and March 2021, and the software was developed using O-Tree. The experiment took about 40 min. All participants received a show-up fee of CNY 10, and the average payment was CNY 33.81.

The experiment consisted of three phases. In the first phase, participants were asked to complete the individualism–collectivism priming task/no priming task. In the second phase, all participants were asked to complete the ultimatum game and the dictator game. In the third phase, all participants completed a risk preference scale and a demographic information questionnaire.

#### 2.2.1. Phase 1. Individualism-Collectivism Priming Tasks 

In Phase 1, 160 participants were primed with a pronoun circling task [12,13] and a group imagination task [18]. Both priming methods have been adopted in prior research and have proven to be appropriate to prime individualism or collectivism culture [6]. Eighty participants in the no-priming condition read two descriptive texts and materials unrelated to priming. 

In the pronoun circling task [12,13], participants read a story about a trip to the countryside. In the individualism condition, the task was to circle personal singular pronouns (I, me, my) in the texts. In the collectivism condition, the task was to circle plural pronouns (we, us, ours) in the texts. All participants were asked to write down the number of pronouns.

In the group imagination task [18], participants were asked to imagine a specific scenario and then describe thoughts from the perspective of the person involved. In the individualism condition, participants were exposed to the individual event, “Someone is playing in a tennis tournament and has made it to the finals. If they win this last match, they will win the championship title as well as a huge trophy.” In the collectivism condition, participants were exposed to the team event, “Someone’s team is playing in a tennis tournament and has made it to the finals. They are representing their team in the final. If they win this last match, their team will win the championship title as well as a huge trophy.” 

#### 2.2.2. Phase 2. Ultimatum Game and Dictator Game

The ultimatum game [9] consists of two players, a proposer and a responder. The proposer receives a sum of money and decides to allocate amount x to the responder. The responder chooses to accept or reject. If the responder accepts, the proposer gets y-x, and the responder gets x; if the responder refuses, both get 0. To ensure that they had understood the rules of the game, subjects had to accurately answer four test questions on the rules of the game before they entered the game.

The standard ultimatum game is a one-round system, which avoids learning effects but has a small sample size. The ultimatum game in our study is a multi-round system, which can enlarge the sample size but may have learning effects and interfere with the experimental results. 

Based on the above, the ultimatum game in this study included 20 rounds (20 rounds are completed in order), with all subjects in rounds 1~10 acting as proposers to make allocation proposals, and all subjects in rounds 11~20 acting as responders to respond to the allocation proposals of the proposers in rounds 1 to 10. In each of the 11-20 rounds, subjects were randomly matched with other subjects in the same treatment to form proposer–responder pairs (pairs rematched each round). Responders in rounds 11 to 20 were randomly matched to proposers in rounds 1 to 10, e.g., round 17 matched round 1, and round 15 matched round 2, and so on. Specifically, subject 1 in round 17 responded to the offer of subject 3 (randomly matched with someone of the subjects 2~8) in round 1, and so on.

To examine the effect of the stakes on the allocation behavior, the stakes in rounds 1~10 were different, and each round was randomly chosen from 100, 200, 300, 400, 500, 600, 700, 800, 900, and 1000 tokens, and was not repeated. Tokens were exchanged at the following rate: 40 tokens = CNY 1. For comparison purposes, all subjects in the same round in rounds 1~10 received the same number of stakes. 

The outcome of each round of the ultimatum game was not fed back to the subjects. Proposers were only able to see their offer pages in rounds 1 to 10 and were not given any additional information. Responders could only see the pages of the proposer’s offer they need to respond to in rounds 11 to 20 and were not informed of the experiment’s results. The computer randomly selected two rounds from rounds 1–20 (one round from rounds 1-10 and one round from rounds 11–20) to determine the experimental payoff in the ultimatum game. Our experimental design expanded the sample size and avoided learning effects on experimental effects as much as possible. 

After completing the ultimatum game, all subjects entered the dictator game. Before entering the dictator game, subjects were asked to review previous individualism-priming or collectivism-priming texts and set the titles for the priming texts to enforce cultural manipulation.

The dictator game [10] consists of two players, a dictator and a responder. The dictator receives a sum of money and decides to allocate amount x to the responder, who cannot reject the offer. The dictator game included 10 rounds (10 rounds are completed in order), and participants played the dictator game as a dictator only (rounds 1~10). The stakes in rounds 1~10 were different and were randomly selected from 10 numbers, as in the ultimatum game. At the end of the dictator game, 1 of the 10 rounds was randomly selected for payment.

#### 2.2.3. Phase 3. Scale and Questionnaire

Besides decisions in the games, we investigated the effects of risk preference and demographic information variables (e.g., gender, and age). Participants were asked to complete the risk preference scale [42] and demographic information questionnaire; see Appendix A for details.

## 3. Results

### 3.1. Manipulation Check

In the pronoun circling task, our manipulation check was the number of pronouns participants clicked on. Following Grossmann and Jowhari [43], we dropped participants from analyses when they clicked fewer than 75% of relevant pronouns. We found that the two subjects that failed the manipulation check for the pronoun circling task were in the collectivism-priming condition.

In the group imagination task, our manipulation check was that participants wrote something—we dropped those who wrote nothing or something irrelevant to the question such as a string of letters (e.g., “aaa”). Two of the subjects that failed the manipulation check for the group imagination task were in the collectivism-priming condition. 

### 3.2. Descriptive Statistics by Group

We excluded four subjects with inadequate initiation: two subjects failed the manipulation check for the pronoun-circling task but passed the manipulation check for the group imagination task; the other two subjects did the opposite. Therefore, we used 236 subjects for data analysis (Collectivism-priming *n* = 76, Individualism-priming *n* = 80, No-priming *n* = 80). As shown in Table 1, proposers and responders in the UG and dictators in the DG all played a game of 10 rounds, so the dependent variables (UG offer, DG offer, and UG accept) of three treatments have 2360 samples in total. The UG accept was significantly different among the three groups. We found that demographic variables other than age did not differ significantly between the three treatments. The average age was 21.58 years, and the average age difference in each group did not exceed one year, so the effect of age was extremely slight. These demographic information variables are included as control variables in the regression analysis later (Table 2, Table 3, Table 4 and Table 5). 

### 3.3. Comparison Analysis of UG Offer and DG Offer

**Figure 1:**
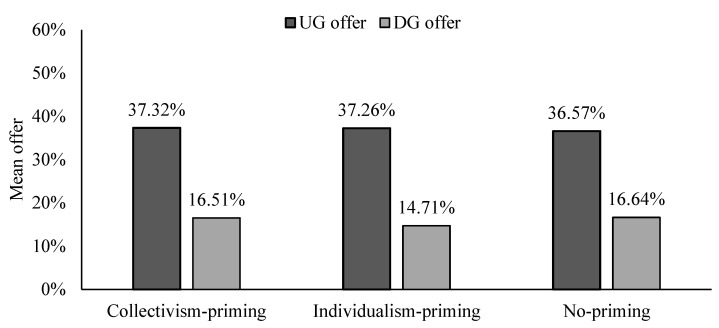
Comparison of mean offer.

**Figure 2:**
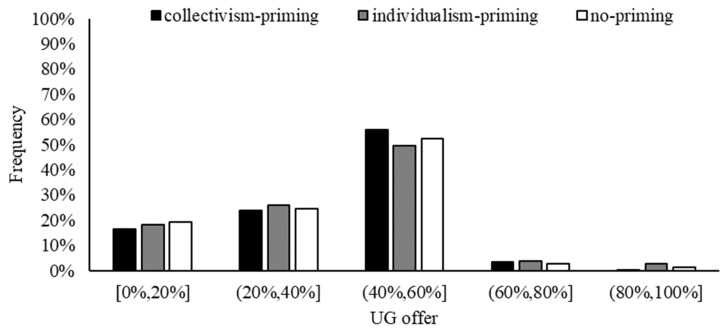
Frequency of UG offers.

We directly compared the differences in the offers of participants under collectivism-priming and individualism-priming conditions. The total sample size of the two treatments is 1560. There is no statistically significant difference in mean UG offer between collectivism-priming and individualism-priming conditions, t (1560) = 0.066, *p* = 0.948 (see Figure 1). As can be seen in Figure 2, most of the UG offers of the samples are mainly concentrated in the 40~60% range. The frequency of UG offers between 40% and 60% is 6.54% higher in the collectivism-priming condition than in the individualism-priming condition, but this difference does not lead to a significant difference overall. If we include explanatory variables for regression analysis, the coefficient of “Collectivism vs. Individualism” is not significant (Table 2, Tobit 1,2). It suggests that priming collectivism (vs. individualism) does not cause a significant difference in participants’ UG offers.

**Figure 3:**
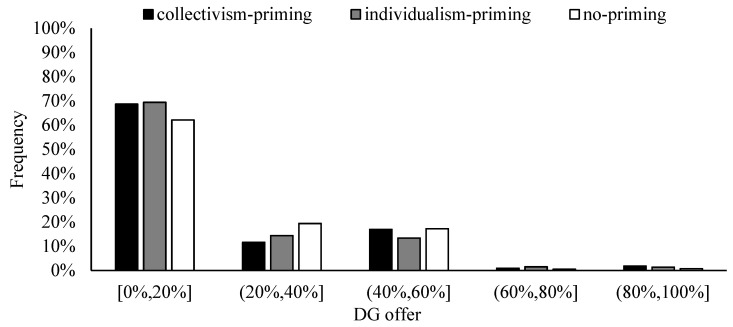
Frequency of DG offers.

However, the mean DG offer of the collectivism-priming and individualism-priming conditions are weakly significantly different (see Figure 1), t (1560) = 1.738, *p* = 0.082. As shown in Figure 3, the DG offer is centrally distributed in the range of 0% to 20%. The frequency of DG offers between 40% and 60% is 3.09% higher in the collectivism-priming condition than in the individualism-priming condition, the 3.09% difference has a vital impact on the overall results. The positive coefficient of the “Collectivism vs. Individualism” indicates that participants have a weakly significantly higher mean DG offer in the collectivism-priming (vs. individualism-priming) condition (Table 2, Tobit 3,4). It indicates that collectivism-priming (vs. individualism-priming) promotes an increase in altruistic allocation behavior, although the effect is weaker.

The comparison between the collectivism-priming/individualism-priming condition and the no-priming condition allows us to identify the effect of a single prime effect on the offers. Because Chinese residents have long been influenced by collectivist values, we predict that participants in the no-primed condition behave more closely to the collectivism-primed condition.

As shown in Figure 1, there is no significant difference between UG offer, collectivism-priming vs. no-priming, t (1560) = 0.878, *p* = 0.380, and individualism-priming vs. no-priming, t (1600) = 0.742, *p* = 0.458. We included all explanatory variables for regression analysis (see Table 3), and the coefficients of “Collectivism vs. No” and “Individualism vs. No” are not statistically significant (Table 3, Tobit 1, 2). This suggests that priming individualism or collectivism does not lead to a significant shift in the UG offer of Chinese participants.

Participants in the individualism-priming (vs. no-priming) condition reported lower mean DG offer (see Figure 1), t (1600) = −1.949, *p* = 0.051; the coefficient of “Individualism vs. No” is significantly negative (Table 3, Tobit 3, 4). Participants in the collectivism-priming condition do not differ significantly from the no-priming condition in DG offer, t (1560) = −0.121, *p* = 0.904 (see Figure 1); the coefficient of “Collectivism vs. No” is not statistically significant (Table 3, Tobit 3, 4). This suggests that the DG offers of the Chinese participants are not affected by the collectivism priming; however, the individualism priming results in the subjects allocating less to others and being more self-interested in the DG.

### 3.4. Comparison Analysis of UG Responders’ Behavior

**Figure 4:**
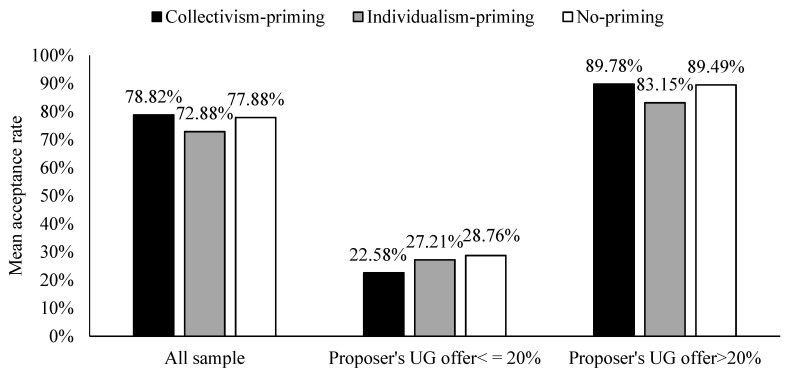
Comparison of mean acceptance rate.

The mean acceptance rates of the two priming conditions are significantly different (see Figure 4), t (1560) = 2.748, *p* = 0.006. If we include explanatory variables for regression analysis, the positive coefficient of the “Collectivism vs. Individualism” indicates that responders accept more offers in the collectivism-priming (vs. individualism-priming) condition (Table 4, Probit 1). Furthermore, we classified the proposer’s offers that were no higher than 20% as extremely unfair allocation behavior and the opposite as non-extremely unfair allocation behavior and performed regression analysis separately (Table 4, Probit 2.3). The sub-sample regressions show that this difference is robust. It shows that responders in the collectivism-priming (vs. individualism-priming) condition have more tolerance for unfair allocation behavior.

As shown in Figure 4, we cannot find significant differences between collectivism-priming and no-priming conditions in the mean acceptance rate, t (1560) = 0.451, *p* = 0.652. Although the coefficient of “Collectivism vs. No” is not significant (Table 4, Probit 1), the mean acceptance rate differed significantly between individualism-priming and no-priming conditions, t (1600) = −2.324, *p* = 0.020. The negative coefficient of the “Individualism vs. No” indicates that responders have significantly less acceptance behavior in the individualism-priming (vs. no-priming) condition (Table 5, Probit 1). We found that the coefficient of “Individualism vs. No” was significantly negative only when the proposer’s UG offer was more than 20% (non-extremely unfair) (Table 5, Probit 2,3). It suggests that the behavior of the responder does not shift after collectivism priming, but the individualism priming results in the responder being less likely to accept the proposer’s non-extreme unfair allocation behavior in the ultimatum game.

## 4. Discussion

In most extant cross-country/cross-culture studies, cross-country differences are attributed to cultural differences without specifying the cultural traits that underlie differences in subjects’ allocation behavior [1,2,19,20,28,29,30,31,32,33,34,35,36,37,44]. Our study focuses on cultural traits in the individualism–collectivism dimension. To study the influence of cultural values on allocation behavior, we experimentally manipulated the individualistic or collectivistic orientation of participants, who then completed ultimatum and dictator games.

### 4.1. Allocation Behavior in the Ultimatum Game

In the ultimatum game, there is no significant difference in the proposers’ mean UG offers in the collectivism-primed (vs. individualism-primed) condition. Although the percentage of participants who offered 40–60% in the collectivism-primed condition is higher (vs. individualism-primed), the difference is not significant overall. The reason for the statistically insignificant difference in UG offers between the two initiation conditions may be that the UG offers are affected by two effects that cancel each other out. Participants in the collectivism-priming condition are more willing to allocate more to the responders, whilst potentially being concerned about allocating less and being rejected by the responders, resulting in no gain for themselves.

However, responders have a higher mean acceptance rate of proposers’ offers in the collectivism-primed (vs. individualism-primed) condition. Our results show that participants are more inclusive of inequitable allocation behavior after collectivism (vs. individualism) initiation. A possible explanation is that participants who reinforce collectivist values are more likely to recognize that what the two players get in total matters more than what one alone gets [40]. Another possible reason is that individuals from collectivist cultures are more likely to try to maintain harmonious relationships and avoid offending others than those from individualist cultures [4,11,45].

### 4.2. Allocation Behavior in the Dictator Game

In the dictator game, participants in the collectivism-primed (vs. individualism-primed) condition had a higher mean DG offer (16.51% vs. 14.71%). Although the difference is relatively small, only less than 2%, considering the low mean DG offer, this effect is also important. It is mainly in the collectivism-primed (vs. individualism-primed) condition that more dictators are willing to allocate 40% to 60% of the stakes to the responders. Triandis [4] includes self-interest in his definition of individualism, following Hofstede [3], who declares selfishness to be an inherent facet of individualism. In a deeply collectivist society, cultural values, such as altruistic generosity and pro-social concerns, are vital sharing motives [46,47,48]. Altruism refers to behavior that benefits others at a personal cost [49], and it plays an essential role in our society. To maintain a harmonious atmosphere in society, people are encouraged to show altruistic behavior toward others. The possible reasons are that people in collectivist cultures perceive that they have a closer relationship with others and are more likely to cooperate and sacrifice their benefits for others than those in individualist cultures [3,11,29,45].

Our study also found that the Chinese samples were not affected by collectivism initiation but exhibited more self-interested behavior and less tolerance for inequitable allocation behavior after individualism initiation. This is in line with previous studies, in which those initiatives consistent with the dominant culture did not have a significant effect, and those that were inconsistent with the dominant culture were more likely to cause a shift in participant behavior [6,13,18].

### 4.3. Limitations and Directions for Future Research

There are some limitations to the present study. First, as an experimental economic study, this research suffers from common problems with existing studies and has a relatively limited sample size. Second, given that a sample of Chinese university students is used for laboratory experiments, whether the sample is representative of society needs to be verified by future studies using additional samples. Third, laboratory-inspired interventions on subjects are only short-term in nature, and it is not possible to determine what the long-term effects of such experimental interventions are. We will continue to extend and refine the relevant findings in future studies. Finally, the present study examined the effects of individualism and collectivism on allocation behavior only from the perspective of two-person pairs. Some studies have shown that collectivists show a more stable tendency toward in-group favoritism than individualists [4,50]. In the future, we will divide the subjects into multi-person groups (e.g., four-person groups) and explore the differences in individual allocation behaviors toward inner and outer group members under the influence of individualism and collectivism [51,52].

## 5. Conclusions

In the current research, we conducted an economics experiment to investigate the effect of individualism and collectivism on allocation behavior. The results show that participants in the collectivism-primed (vs. individualism-primed) condition have more altruistic allocation behavior in the dictator game and more tolerance for unequal allocation behavior in the ultimatum game. Our research also shows that collectivism initiation has a relatively weak effect on Chinese participants, and individualistic initiation leads to a shift in the allocation behavior of Chinese participants in a direction opposite to that of collectivism.

## Figures and Tables

**Table 1:** Descriptive statistics by group.

Variables	Collectivism- Priming			Individualism- Priming			No-Priming			ANOVA *p*-Values
	N	Mean	SD	N	Mean	SD	N	Mean	SD	
UG offer (0%~100%)	760	37.32%	0.16	800	37.26%	0.20	800	36.57%	0.18	0.652
DG offer (0%~100%)	760	16.51%	0.21	800	14.71%	0.20	800	16.64%	0.20	0.101
UG accept (accept = 1, reject = 0)	760	78.82%	0.41	800	72.88%	0.44	800	77.88%	0.42	0.011
Gender (male = 1, female = 0)	76	46.05%	0.50	80	50.00%	0.50	80	48.75%	0.50	0.883
Age	76	21.20	1.46	80	21.41	1.56	80	22.10	1.76	0.001
Risk preference(1~11)	76	5.32	1.27	80	5.08	1.35	80	4.93	1.13	0.148
Party member(yes = 1, no = 0)	76	0.11	0.31	80	0.15	0.36	80	0.13	0.33	0.705
Urban(urban = 1, other = 0)	76	0.54	0.50	80	0.61	0.49	80	0.56	0.50	0.643
Part-time job(yes = 1, no = 0)	76	0.76	0.43	80	0.70	0.46	80	0.83	0.38	0.180
Mother’s education(1~ 6)	76	2.74	1.63	80	2.93	1.68	80	2.93	1.65	0.718
Father’s education(1~ 6)	76	3.07	1.56	80	3.26	1.57	80	3.23	1.65	0.719
Family income (1~6)	76	2.66	1.00	80	2.74	1.23	80	2.69	0.88	0.891

Notes: Mother’s education and father’s education included six categories: 1 = elementary school and below; 2 = junior high school; 3 = high school; 4 = technical secondary school; 5 = junior college; 6 = University and above. Family income is divided into six levels: 1 = less than 50,000; 2 = 50,000–100,000; 3 = 100,000–250,000; 4 = 250,000–500,000; 5 = 500,000-1 million; 6 = more than 1 million.

**Table 2:** Regression analysis of two treatments of offer.

	Dependent Variables: UG Offer		Dependent Variables: DG Offer	
	Tobit 1	Tobit 2	Tobit 3	Tobit 4
Collectivism vs. Individualism	−0.001	0.005	0.050 *	0.036
	(0.017)	(0.015)	(0.026)	(0.025)
Log(stake)	−0.002	−0.002	−0.013 *	−0.013 **
	(0.005)	(0.005)	(0.007)	(0.007)
Round	0.001	0.001	−0.005 ***	−0.005 ***
	(0.001)	(0.001)	(0.002)	(0.002)
Gender		0.036		−0.065
		(0.025)		(0.046)
Age		-0.010		−0.015
		(0.011)		(0.012)
Risk preference		0.008		0.031 **
		(0.009)		(0.015)
Session dummy	Yes	Yes	Yes	Yes
Other control variables *	No	Yes	No	Yes
Constant	0.372 ***	0.470 *	0.254 ***	0.462
	(0.045)	(0.244)	(0.062)	(0.290)
Sample size	1560	1560	1560	1560
Participants	156	156	156	156

Notes: Tobit regression analysis, *, **, *** significant at 10, 5, 1 percent level. Robust standard errors in parentheses, clustered at subgroup (group of 8 participants). Considering that there are two treatments in each session, 8 subjects in the same treatment are randomly paired in each round during the game, and there are a total of 20 subgroups in 10 sessions, we clustered the robust standard errors to the subgroup level. * Other control variables included Party member, Urban, Part-time job, Mother’s education (dummy variables), Father’s education (dummy variables), and Family income (dummy variables). Excluding the four subjects with inadequate initiation, we included 156 subjects in the regression analysis with a sample size of 1560 for 10 rounds. The results before exclusion are generally consistent, as described in the supplementary material.

**Table 3:** Regression analysis of three treatments of offer.

	Dependent Variables: UG Offer		Dependent Variables: DG Offer	
	Tobit 1	Tobit 2	Tobit 3	Tobit 4
Collectivism vs. No	0.006	0.010	0.005	−0.003
	(0.015)	(0.015)	(0.023)	(0.030)
Individualism vs. No	0.006	0.010	−0.044 *	−0.043 **
	(0.015)	(0.017)	(0.023)	(0.021)
Log(stake)	−0.010 **	−0.010 **	−0.014 ***	−0.014 ***
	(0.005)	(0.005)	(0.005)	(0.005)
Round	0.001	0.001	−0.004 ***	−0.004 ***
	(0.001)	(0.001)	(0.001)	(0.001)
Gender		0.017		−0.056 *
		(0.022)		(0.033)
Age		-0.001		−0.012
		(0.010)		(0.011)
Risk preference		0.000		0.007
		(0.008)		(0.013)
Session dummy	Yes	Yes	Yes	Yes
Other control variables *	No	Yes	No	Yes
Constant	0.422 ***	0.376 *	0.267 ***	0.470 *
	(0.037)	(0.220)	(0.053)	(0.274)
Sample size	2360	2360	2360	2360
Participants	236	236	236	236

Notes: Tobit regression analysis, *, **, *** significant at 10, 5, 1 percent level. Robust standard errors in parentheses, clustered at subgroup (group of 8 participants). Considering that there are three treatments in each session, 8 subjects in the same treatment are randomly paired in each round during the game, and there are a total of 30 subgroups in 10 sessions, we clustered the robust standard errors to the subgroup level. * Other control variables included Party member, Urban, Part-time job, Mother’s education (dummy variables), Father’s education (dummy variables), and Family income (dummy variables). Excluding the four subjects with inadequate initiation, we included 236 subjects in the regression analysis with a sample size of 2360 for the 10 rounds. The results before exclusion are generally consistent, as described in the supplementary material.

**Table 4:** Regression analysis of two treatments of responders’ behavior.

Dependent Variables: UG Accept (Accept = 1)	All Sample	Proposer’s UG Offer< = 20%	Proposer’s UG Offer>20%
	Probit 1	Probit 2	Probit 3
Collectivism vs. Individualism	0.324 ***	2.876 ***	0.432 ***
	(0.086)	(0.583)	(0.101)
Proposer’s UG offer	7.648 ***	36.648 ***	7.208 ***
	(0.729)	(5.702)	(1.265)
Own UG offer ^1^	−2.922 ***	−12.681 ***	−2.394 ***
	(0.384)	(3.410)	(0.287)
Log(stake)	0.303 ***	0.633	0.316 ***
	(0.053)	(0.449)	(0.057)
Round	0.013	−0.063	0.018
	(0.017)	(0.121)	(0.022)
Gender	0.084	2.476 **	−0.029
	(0.165)	(1.154)	(0.166)
Age	−0.003	0.991 **	−0.048
	(0.056)	(0.464)	(0.063)
Risk preference	−0.208 ***	−1.529 **	−0.174 ***
	0.324 ***	(0.644)	(0.051)
Session dummy	Yes	Yes	Yes
Other control variables ^2^	Yes	Yes	Yes
Constant	−1.818	−21.321 ***	−1.130
	(1.236)	(7.822)	(1.427)
Sample size	1560	271	1289
Participants	156		

Notes: Probit regression analysis, **, *** significant at 5, 1 percent level. Robust standard errors in parentheses, clustered at subgroup (group of 8 participants). Considering that there are two treatments in each session, 8 subjects in the same treatment are randomly paired in each round during the game, and there are a total of 20 subgroups in 10 sessions, we clustered the robust standard errors to the subgroup level. ^1^ Own UG offer is the UG offer when they were faced with the same reward. ^2^ Other control variables include Party member, Urban, Part-time job, Mother’s education (dummy variables), Father’s education (dummy variables), and Family income (dummy variables). Excluding the four subjects with inadequate initiation, we included 156 subjects in the regression analysis with a sample size of 1560 for the 10 rounds. The results before exclusion are generally consistent, as described in the supplementary material.

**Table 5:** Regression analysis of three treatments of responders’ behavior.

Dependent Variables: UG Accept (Accept = 1)	All Sample	Proposer’s UG Offer< = 20%	Proposer’s UG Offer > 20%
	Probit 1	Probit 2	Probit 3
Collectivism vs. No	−0.037	0.170	0.012
	(0.118)	(0.315)	(0.109)
Individualism vs. No	−0.242 *	−0.004	−0.302 **
	(0.134)	(0.230)	(0.117)
Proposer’s UG offer	7.449 ***	12.683 ***	7.544 ***
	(0.488)	(1.755)	(1.037)
Own UG offer ^1^	−3.040 ***	−5.469 ***	−2.328 ***
	(0.337)	(0.728)	(0.285)
Log(stake)	0.183 ***	0.193	0.186 ***
	(0.056)	(0.137)	(0.062)
Round	−0.004	0.004	−0.009
	(0.016)	(0.026)	(0.020)
Gender	−0.025	−0.063	−0.024
	(0.118)	(0.247)	(0.131)
Age	0.037	0.116	0.010
	(0.038)	(0.086)	(0.044)
Risk preference	−0.070	−0.159	−0.057
	(0.046)	(0.122)	(0.045)
Session dummy	Yes	Yes	Yes
Other control variables ^2^	Yes	Yes	Yes
Constant	−2.081 **	−4.272 *	−1.762
	(0.995)	(2.408)	(1.157)
Sample size	2360	424	1936
Participants	236		

Notes: Probit regression analysis, *, **, *** significant at 10, 5, 1 percent level. Robust standard errors in parentheses, clustered at subgroup (group of 8 participants). Considering that there are three treatments in each session, 8 subjects in the same treatment are randomly paired in each round during the game, and there are a total of 30 subgroups in 10 sessions, we clustered the robust standard errors to the subgroup level. ^1^ Own UG offer is the UG offer when they were faced with the same reward. ^2^ Other control variables included Party member, Urban, Part-time job, Mother’s education (dummy variables), Father’s education (dummy variables), and Family income (dummy variables). Excluding the four subjects with inadequate initiation, we included 236 subjects in the regression analysis with a sample size of 2360 for the 10 rounds. The results before exclusion are generally consistent, as described in the supplementary material.

## Data Availability

The data that support the findings of this study are available on request from the corresponding author.

## Supplementary Materials

The following supporting information can be downloaded at: https://www.mdpi.com/article/10.3390/bs13020169/s1, Table S1: Descriptive Statistics by group; Figure S1: Comparison of mean offer; Figure S2: Frequency of UG offers; Figure S3. Frequency of DG offers; Table S2. Regression analysis of two treatments of offer; Table S3. Regression analysis of three treatments of offer; Figure S4. Comparison of mean acceptance rate; Table S5. Regression analysis of three treatments of responders’ behavior.

## Appendix A. Risk Preference Measurement

Please answer the following 11 questions, each of which involves choosing between options A and B. As Table A1, option A is a lottery ticket, and option B is a definite gain. If you choose option A, your return is uncertain, with a 50% probability that you will receive CNY 0 and 50% probability that you will receive CNY 10; if you choose option B, you will receive the corresponding value of the return.

**Table A1:** Risk preference scale.

	A	B	Your Choice
1	50% probability get CNY 0; 50% probability get CNY 10	Determine to get CNY 0	
2	50% probability get CNY 0; 50% probability get CNY 10	Determine to get CNY 1	
3	50% probability get CNY 0; 50% probability get CNY 10	Determine to get CNY 2	
4	50% probability get CNY 0; 50% probability get CNY 10	Determine to get CNY 3	
5	50% probability get CNY 0; 50% probability get CNY 10	Determine to get CNY 4	
6	50% probability get CNY 0; 50% probability get CNY 10	Determine to get CNY 5	
7	50% probability get CNY 0; 50% probability get CNY 10	Determine to get CNY 6	
8	50% probability get CNY 0; 50% probability get CNY 10	Determine to get CNY 7	
9	50% probability get CNY 0; 50% probability get CNY 10	Determine to get CNY 8	
10	50% probability get CNY 0; 50% probability get CNY 10	Determine to get CNY 9	
11	50% probability get CNY 0; 50% probability get CNY 10	Determine to get CNY 10	

Notes: The number of risk options is a measure of the subject’s risk preference; the higher the number of risk options, the greater the risk preference.
