# Supplementary material for: Individualism, Collectivism, and Allocation Behavior: Evidence from the Ultimatum Game and Dictator Game
Source: Behav Sci (Basel). 2023 Feb 14;13(2):169. doi: 10.3390/bs13020169 (PMC9951955; doi:10.3390/bs13020169)
Supplement: Supplementary file 1 [file behavsci-13-00169-s001.zip › behavsci-2103302-supplementary.pdf]

Supplemental Material

In the supplemental material, we reported the results of the analysis of the 240 subjects（2400 samples for 10 rounds) which were generally consistent with the results of excluding the 4 subjects（40 samples) with inadequate initiation.

Table S1. Descriptive Statistics by group.

Variables	Collectivism-priming			Individualism-priming			No-priming			ANOVA P-values
	N	Mean	SD	N	Mean	SD	N	Mean	SD	
UG offer (0%~100%)	800	37.54%	0.16	800	37.26%	0.20	800	36.57%	0.18	0.652
DG offer (0%~100%)	800	16.70%	0.21	800	14.71%	0.20	800	16.64%	0.20	0.101
UG accept (accept=1,reject=0)	800	78.88%	0.41	800	72.88%	0.44	800	77.88%	0.42	0.011
Gender (male=1, female=0)	80	48.75%	0.50	80	50.00%	0.50	80	48.75%	0.50	0.883
Age	80	21.23	1.44	80	21.41	1.56	80	22.10	1.76	0.001
Risk preference（1~ 11）	80	5.35	1.26	80	5.08	1.35	80	4.93	1.13	0.148
Party member（yes=1, no=0）	80	0.10	0.30	80	0.15	0.36	80	0.13	0.33	0.705
Urban（urban=1, other=0）	80	0.54	0.50	80	0.61	0.49	80	0.56	0.50	0.643
Part-time job（yes=1, no=0）	80	0.78	0.42	80	0.70	0.46	80	0.83	0.38	0.180
Mother's education（1~ 6）	80	2.69	1.61	80	2.93	1.68	80	2.93	1.65	0.718
Father's education（1~ 6）	80	3.03	1.53	80	3.26	1.57	80	3.23	1.65	0.719
Family income （1~ 6）	80	2.69	1.01	80	2.74	1.23	80	2.69	0.88	0.891

Notes: Mother's education and Father's education included six types,1 = elementary school and below; 2 = junior high school; 3=high school; 4=technical secondary school; 5 = junior college; 6 = University and above. Family income is divided into six levels, 1=less than 50,000; 2=50,000-100,000; 3=100,000-250,000; 4=250,000-500,000; 5 = 500,000-1 million; 6 = more than 1 million.

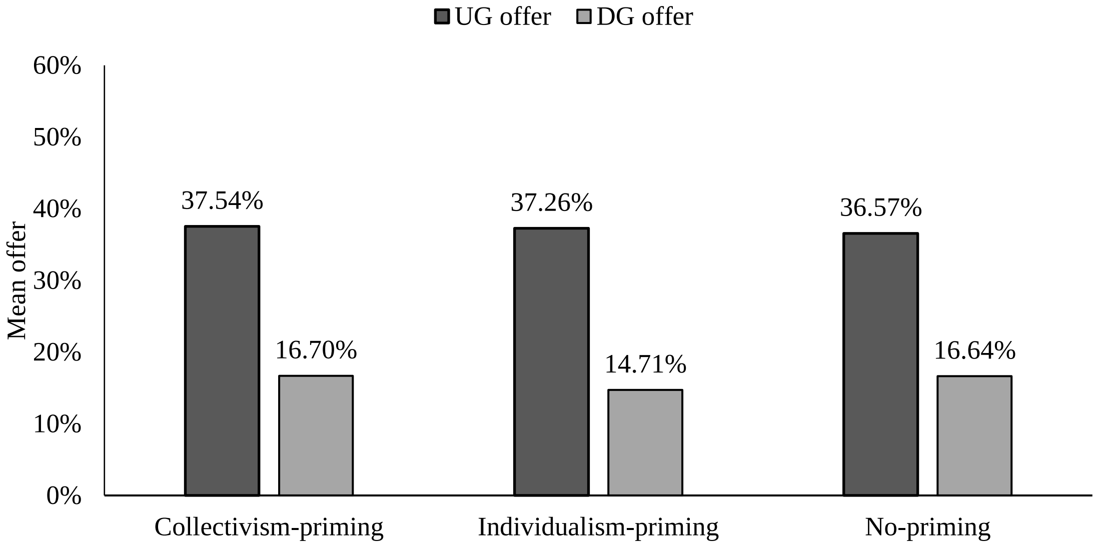

Figure S1. Comparison of mean offer in the full sample.

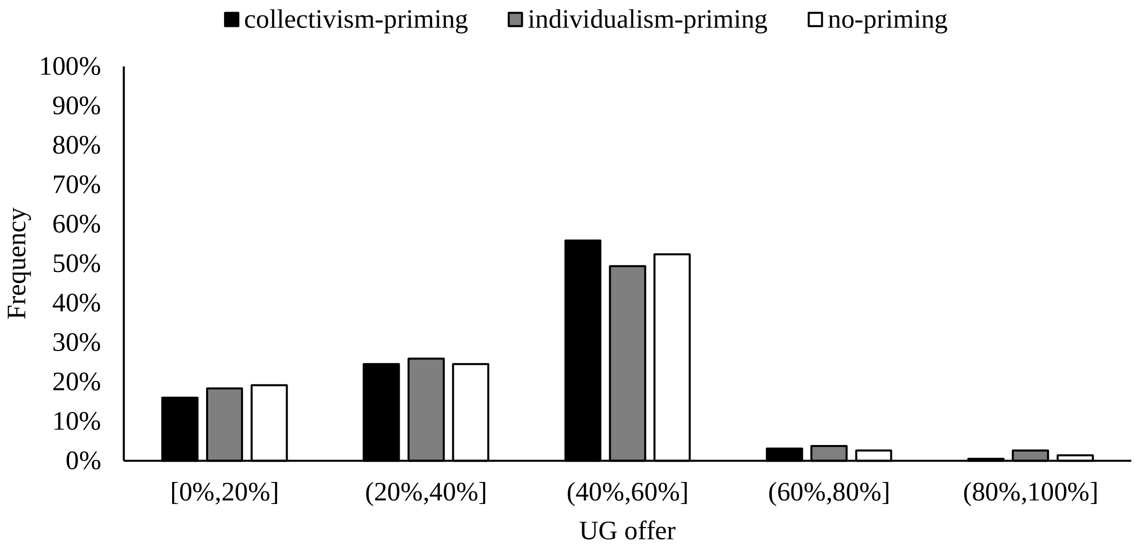

Figure S2. Frequency of UG offers in the full sample.

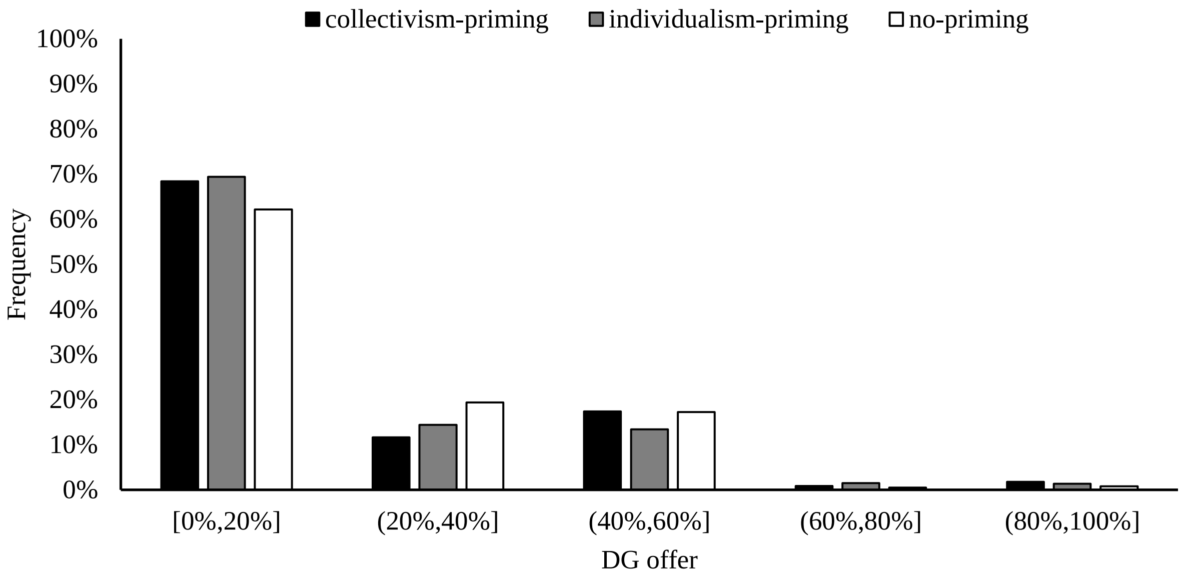

Figure S3. Frequency of DG offers in the full sample.

Table S2. Regression analysis of two treatments of offer.

	Dependent variables: UG offer		Dependent variables: DG offer	
	**Tobit 1**	**Tobit 2**	**Tobit 3**	**Tobit 4**
Collectivism vs. Individualism	0.004	0.007	0.055**	0.041
	(0.017)	(0.016)	(0.026)	(0.026)
Log(stake)	-0.004	-0.004	-0.012*	-0.012*
	(0.005)	(0.005)	(0.006)	(0.006)
Round	0.002	0.002	-0.005***	-0.005***
	(0.001)	(0.001)	(0.002)	(0.002)
Gender		0.039		-0.057
		(0.024)		(0.047)
Age		-0.010		-0.013
		(0.010)		(0.012)
Risk preference		0.007		0.027*
		(0.009)		(0.014)
Session dummy	Yes	Yes	Yes	Yes
Other control variables*	No	Yes	No	Yes
Constant	0.378***	0.466**	0.245***	0.419
	(0.046)	(0.234)	(0.060)	(0.289)
Sample size	1600	1600	1600	1600
Participants	160	160	160	160

Notes: Tobit regression analysis, *, **, *** significant at 10, 5, 1 percent level. Robust standard errors in parentheses, clustered at subgroup (group of 8 participants). Considering that there are two treatments in each session, 8 subjects in the same treatment are randomly paired in each round during the game, and there are a total of 20 subgroups in 10 sessions, we clustered the robust standard errors to the subgroup level. *Other control variables included Party member, Urban, Part-time job, Mother's education (dummy variables), Father's education (dummy variables), Family income (dummy variables).

Table S3. Regression analysis of three treatments of offer.

	Dependent variables: UG offer		Dependent variables: DG offer	
	**Tobit 1**	**Tobit 2**	**Tobit 3**	**Tobit 4**
Collectivism vs. No	0.010	0.013	0.010	0.003
	(0.015)	(0.015)	(0.022)	(0.029)
Individualism vs. No	0.006	0.011	-0.044*	-0.041*
	(0.016)	(0.017)	(0.023)	(0.021)
Log(stake)	-0.011**	-0.011**	-0.013***	-0.013***
	(0.005)	(0.005)	(0.005)	(0.005)
Round	0.001	0.001	-0.004***	-0.004***
	(0.001)	(0.001)	0.010	0.003
Gender		0.020		-0.053*
		(0.021)		(0.032)
Age		-0.000		-0.011
		(0.010)		(0.011)
Risk preference		0.000		0.006
		(0.008)		(0.013)
Session dummy	Yes	Yes	Yes	Yes
Other control variables*	No	Yes	No	Yes

Constant	0.426***<br>(0.037)	0.366*<br>(0.215)	0.261***<br>(0.052)	0.451*<br>(0.268)
Sample size	2400	2400	2400	2400
Participants	240	240	240	240

Notes: Tobit regression analysis,*, **, *** significant at 10, 5, 1 percent level. Robust standard errors in parentheses, clustered at subgroup (group of 8 participants). Considering that there are three treatments in each session, 8 subjects in the same treatment are randomly paired in each round during the game, and there are a total of 30 subgroups in 10 sessions, we clustered the robust standard errors to the subgroup level. *Other control variables included Party member, Urban, Part-time job, Mother's education (dummy variables), Father's education (dummy variables), Family income (dummy variables).

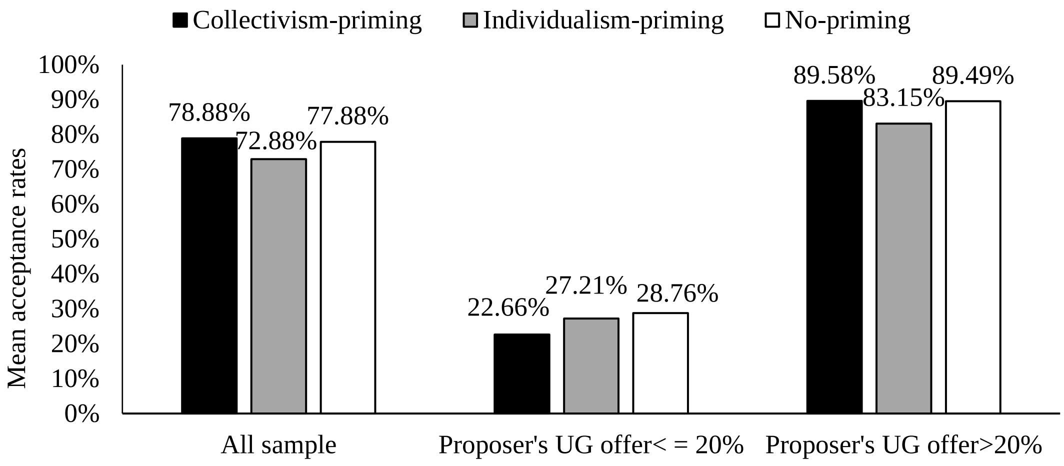

Figure S4. Comparison of mean acceptance rate in the full sample.

Table S4. Regression analysis of two treatments of responders' behavior.

**Dependent variables: UG accept (accept=1)**	**All sample**	**Proposer's UG offer< = 20%**	**Proposer's UG offer>20%**
	**Probit 1**	**Probit 2**	**Probit 3**
Collectivism vs. Individualism	0.320***<br>(0.084)	2.068***<br>(0.566)	0.434***<br>(0.100)
Proposer's UG offer	7.672***<br>(0.712)	29.167***<br>(5.726)	7.293***<br>(1.227)
Own UG offer[1]	-2.978***<br>(0.386)	-12.158***<br>(3.295)	-2.462***<br>(0.298)
Log(stake)	0.283***<br>(0.058)	0.421<br>(0.391)	0.301***<br>(0.059)
Round	0.014<br>(0.017)	-0.076<br>(0.102)	0.021<br>(0.021)
Gender	0.080<br>(0.155)	2.050**<br>(0.921)	-0.026<br>(0.155)
Age	-0.000<br>(0.058)	0.858*<br>(0.461)	-0.041<br>(0.065)
Risk preference	-0.194***<br>(0.054)	-1.508**<br>(0.588)	-0.157***<br>(0.048)
Session dummy	Yes	Yes	Yes
Other control variables[2]	Yes	Yes	Yes
Constant	-1.786<br>(1.275)	-15.598*<br>(7.959)	-1.284<br>(1.444)

Sample size	1600	275	1325
Participants	160		

Notes: Probit regression analysis, *, **, *** significant at 10, 5, 1 percent level. Robust standard errors in parentheses, clustered at subgroup (group of 8 participants). Considering that there are two treatments in each session, 8 subjects in the same treatment are randomly paired in each round during the game, and there are a total of 20 subgroups in 10 sessions, we clustered the robust standard errors to the subgroup level. [1]Own UG offer is the UG offer when they faced with the same reward. [2]Other control variables included Party member, Urban, Part-time job, Mother's education (dummy variables), Father's education (dummy variables), Family income (dummy variables).

Table S5. Regression analysis of three treatments of responders' behavior.

**Dependent variables: UG accept (accept=1)**	**All sample**	**Proposer's UG offer<=20%**	**Proposer's UG offer>20%**
	**Probit 1**	**Probit 2**	**Probit 3**
Collectivism vs. No	-0.031	0.111	0.023
	(0.116)	(0.297)	(0.105)
Individualism vs. No	-0.240*	-0.023	-0.295**
	(0.133)	(0.226)	(0.115)
Proposer's UG offer	7.479***	12.201***	7.604***
	(0.476)	(1.700)	(1.006)
Own UG offer[1]	-3.064***	-5.538***	-2.363***
	(0.336)	(0.746)	(0.286)
Log(stake)	0.172***	0.161	0.179***
	(0.057)	(0.140)	(0.062)
Round	-0.003	0.002	-0.006
	(0.015)	(0.027)	(0.019)
Gender	-0.025	-0.070	-0.026
	(0.113)	(0.240)	(0.122)
Age	0.038	0.107	0.013
	(0.039)	(0.083)	(0.044)
Risk preference	-0.067	-0.178	-0.051
	(0.047)	(0.123)	(0.044)
Session dummy	Yes	Yes	Yes
Other control variables[2]	Yes	Yes	Yes
Constant	-2.066**	-3.576	-1.837
	(0.996)	(2.325)	(1.151)
Sample size	2400	428	1972
Participants	240		

Notes: Probit regression analysis, *, **, *** significant at 10, 5, 1 percent level. Robust standard errors in parentheses, clustered at subgroup (group of 8 participants). Considering that there are three treatments in each session, 8 subjects in the same treatment are randomly paired in each round during the game, and there are a total of 30 subgroups in 10 sessions, we clustered the robust standard errors to the subgroup level. [1]Own UG offer is the UG offer when they faced with the same reward. [2]Other control variables included Party member, Urban, Part-time job, Mother's education (dummy variables), Father's education (dummy variables), Family income (dummy variables).
